# Celastrol and Triptolide Suppress Stemness in Triple Negative Breast Cancer: Notch as a Therapeutic Target for Stem Cells

**DOI:** 10.3390/biomedicines9050482

**Published:** 2021-04-28

**Authors:** Prabhu Ramamoorthy, Prasad Dandawate, Roy A. Jensen, Shrikant Anant

**Affiliations:** 1 Department of Cancer Biology, University of Kansas Medical Center, Kansas City, KS 66160, USA; pramamoorthy@kumc.edu (P.R.); pdandawate@kumc.edu (P.D.); 2 Department of Pathology and Laboratory Medicine, University of Kansas Medical Center, Kansas City, KS 66160, USA

**Keywords:** mammospheres, DCLK1, ALDH1, γ-secretase, presenilin, nicastrin

## Abstract

Triple negative breast cancer (TNBC) is observed in ~15% of breast cancers and results in poor survival and increased distant metastases. Within the tumor are present a small portion of cancer stem cells that drive tumorigenesis and metastasis. In this study, we aimed to elucidate whether the two natural compounds, celastrol and triptolide, inhibit stemness in TNBC. MDA-MB-231, BT20, and a patient-derived primary cells (PD-TNBC) were used in the study. Mammosphere assay was performed to assess the stemness. Both celastrol and triptolide treatment suppressed mammosphere formation. Furthermore, the compound suppressed expression of cancer stem cell marker proteins DCLK1, ALDH1, and CD133. Notch signaling plays a critical role in stem cells renewal. Both celastrol or triptolide reduced Notch -1 activation and expression of its downstream target proteins HES-1 and HEY-1. However, when NICD 1 was ectopically overexpressed in the cells, it partially rescued proliferation and mammosphere formation of the cells, supporting the role of notch signaling. Together, these data demonstrate that targeting stem cells and the notch signaling pathway may be an effective strategy for curtailing TNBC progression.

## 1. Introduction

Breast cancer is the second leading cause of cancer-related deaths among women worldwide. Although significant progress has been made in reducing breast cancer mortality rates due to advancements in the diagnosis and development of novel radiation and targeted chemotherapies, it remains a significant cause of morbidity and mortality [1]. Chemotherapy regimens have been designed for specific breast cancer subtypes based on their gene expression profile and mutational status. While hormone receptor-positive luminal A and luminal B subtypes are generally eligible for treatment with drugs such as tamoxifen [2], human epidermal growth factor receptor 2 (Her2)-enriched subtypes are treated with drugs such as trastuzumab and pertuzumab [3]. On the other hand, in triple-negative breast cancer (TNBC), where the tumor cells lack expression of estrogen or progesterone receptors and Her2, targeted therapies to these receptors do not work, making cytotoxic chemotherapy the preferred therapeutic option. Hence, patients that have TNBC or a cancer that lacks BRCA1 expression are treated with chemotherapy and poly (ADP-ribose) polymerase (PARP) inhibitors [4]. Unfortunately, in spite of showing initial response to these therapies, there is often tumor relapse following the development of therapeutic resistance. Hence, the need for identifying molecular targets that are amenable to novel therapeutic development.

Cancer stem cells (CSCs) have indefinite proliferative potential because of their ability to self-renew, and this results in tumor invasion and migration, heterogeneity, and therapy resistance. Hence, tumors that encode a stemness-like phenotype are more aggressive and exhibit a poorer prognosis [5]. Earlier studies have demonstrated that CSCs in breast cancer (BCSC) are marked by various proteins including DCLK1, aldehyde dehydrogenase (Aldh1), CD44^high/^CD24^low^, and CD133 [6,7,8]. Furthermore, studies have demonstrated that mammosphere cultures can be used to enrich for BCSCs [9]. Interestingly, the gene expression pattern seen in these mammospheres, but not in 2D cultures relates closely to what is seen in clinical samples [10]. Several stem cell-related pathways are also active in BCSCs including Notch, Hedgehog, and Wnt, which defines and enables stemness in these cells [11].

Natural compounds are increasingly being recognized as promising anticancer agents, and it is thought that they may have the potential to reduce morbidity and mortality by extending the time it takes for cancer development and progression [12]. The plant Trypterygium wilfordii, known as the Thunder God of Vine in traditional Chinese medicine, has been used for centuries against inflammation and cancer [13,14]. Celastrol and triptolide are the major terpenoids isolated from this plant and have been shown to inhibit HSP90 function. Celastrol and triptolide have been shown to enhance cancer chemosensitivity and inhibit cancer cell growth [13]. The compounds have also been shown to induce apoptosis in osteosarcoma, lung cancer, esophageal cancer, and prostate cancer [14,15,16,17,18,19,20,21,22]. Recently, celastrol was shown to induce apoptosis in TNBC cells, the mechanism thought to be by inducing mitochondrial dysfunction and suppressing the PI3K/Akt signaling pathway [14]. However, their role in affecting stemness has not been determined.

In this study, we have determined that both celastrol and triptolide inhibit mammosphere cultures of TNBC cells. Moreover, triptolide demonstrated higher potency in inhibiting stem cells in the mammosphere cultures by targeting the Notch signaling pathway.

## 2. Materials and Methods

### 2.1. Cells and Reagents

Established TNBC cell lines BT20 and MDA-MB-231 were obtained from ATCC and grown in DMEM containing 10% heat-inactivated fetal bovine serum (Sigma-Aldrich, St. Louis, MO, USA) and 1% antibiotic-antimycotic solution (Mediatech Inc, Manassas, VA, USA) at 37 °C in a humidified atmosphere of 5% CO_2_. Celastrol and triptolide were purchased from InvivoGen (San Diego, CA, USA), and N-[N-(3,5-Difluorophenacetyl)-L-alanyl]-S-phenylglycinet-butyl ester (DAPT) was purchased from Sigma-Aldrich (St. Louis, MO, USA).

#### 2.1.1. Patient-Derived Triple Negative Breast Cancer isolation

Deidentified human tumor tissue samples were obtained from the University of Kansas Cancer Center Biospecimen Repository Core Facility. The samples were obtained from patients following written consent. Primary cells were isolated from the tissue as described [23]. Briefly, tumor tissue was washed with PBS and minced into smaller pieces. Then collagenase (1 mg/mL) was added to the minced tissue and incubated for 10 min. Tissue was mixed well using a pipette to derive a single-cell suspension. Collagenase was inactivated by 5% FBS and centrifuged for 3 min at 1500 rpm. Then the pellet was suspended with a special medium containing DMEM, which contained rhEGF (20 ng/mL), rhFGF (20 ng/mL), 1× B27, VEGF (10 ng/mL), and 2% FBS, and was plated in a 100 mM petridish. After 5–10 days, cells were expanded using DMEM containing 10% FBS and used for subsequent experiments.

#### 2.1.2. Spheroid Assay

For mammosphere growth, cells were plated in ultra-low (Corning) tissue culture dishes at a density of 100–5000 cells/mL in a serum-free mammary epithelium basal medium (Lonza, Inc., Morristown, NJ) supplemented with B27 (Invitrogen, Carlbad, CA, USA), 1% antibiotic-antimycotic, 5 μg/mL insulin, 1 μg/mL hydrocortisone, 4 μg/mL gentamicin, 20 ng/mL EGF (Sigma-Aldrich, St. Louis, MO, USA)), 20 ng/mL basic fibroblast growth factor (Sigma-Aldrich, St. Louis, MO, USA), and 1:25,000,000 β-mercaptoethanol (Sigma-Aldrich, St. Louis, MO, USA). Varying concentrations of celastrol and triptolide were added to the primary culture, whereas the second and third passages were grown in the absence of the drug. The number of mammospheres was counted under a Nikon eclipse microscope and the photos were acquired with NIS elements. For proliferation assay, 5000 cells were seeded on to 96-well plates and allowed to attach and grow overnight, following which they were treated with celastrol or triptolide. Analysis of cell proliferation was performed by hexosaminidase assay as previously described [24]. IC50 concentration was calculated using GraphPad Prism5. In all studies, the IC50 concentration was used unless mentioned otherwise.

#### 2.1.3. Western Blot Analysis

Cells and spheroids lysates were prepared, and an equal concentration of protein was loaded to polyacrylamide gel electrophoresis and blotted onto Immobilon-*p* polyvinylidene difluoride membranes (Millipore, Bedford, MA, USA). Antibodies (Table 1 were purchased from Cell Signaling Technology (Beverly, MA, USA), Santa Cruz Biotechnology Inc (Santa Cruz, CA, USA), Mitenyl Biotec (Auburn, CA, USA), BD Biosciences (San Jose, CA, USA), and Abcam (Cambridge, MA, USA), and specific proteins were detected by the enhanced chemiluminescence system (GE Healthcare, Piscataway, NJ, USA). The list of antibodies is shown in Table 1.

#### 2.1.4. Flow Cytometric Analyses

Cell or spheroids treated for 24 h with celastrol (0.5 µM) or triptolide (5 nM) were trypsinized to isolate single cells. Unfixed and non-permeabilized cells were labeled using phycoerythrin-conjugated DCLK1 or phycoerythrin-conjugated CD133. Alternatively, fixed and permeabilized cells were labeled with phycoerythrin-conjugated Aldh1. The samples were analyzed using the Accuri C6 flow cytometer and analyzed by using CFlow Plus software (BD Biosciences).

#### 2.1.5. Plasmid and Transfection

NICD overexpression was performed as described [25] in BT20 cells. p3XFlagNICD1 or p3XFLAG-CMV-7 (an empty vector) plasmids (Addgene Inc., Cambridge, MA, USA) were transfected into the cells. Subsequently, the cells were grown as 2D and 3D cultures and treated with celastrol and triptolide.

#### 2.1.6. Molecular Docking Protocol

The AutoDock Vina docking software [26] was used to evaluate the interaction of celastrol and triptolide with Presenelin-1 (PDB ID: 2KR6) and Nicastrin (PDB ID: 4R12) [27]. For docking analysis, we designed the 3D-grid around the catalytic residue ASP385 for Presenilin 1 and employed grid parameters reported by Pal et al. for Nicastrin [28]. A grid center co-ordinate containing grid box spacing of 1.0 Å and 40 × 40 × 40-point size was used. The default parameters of the AutoDock Vina tools were used for proteins and compounds preparation. We further added Total Kollman and Gasteiger charges to proteins before docking, and the Lamarckian generic algorithm was used to predict top protein-compounds conformations. About 10 conformations for each protein–compound complex was evaluated. The most stable conformation was selected based on the lowest binding energy and the number of hydrogen bonds. The protein–compound complexes were studied and visualized on April 13, 2021 using Pymol (https://pymol.org/2/ (accessed on 13 April 2021)) software [29].

#### 2.1.7. Statistical Analysis

All values are expressed as the mean 3 ± SD. Data were analyzed using an unpaired two-tailed t-test. *p*-value of less than 0.05 was considered statistically significant.

## 3. Results

### Natural Compound Celastrol and Triptolide Inhibit Spheroid Growth

**Figure 1:**
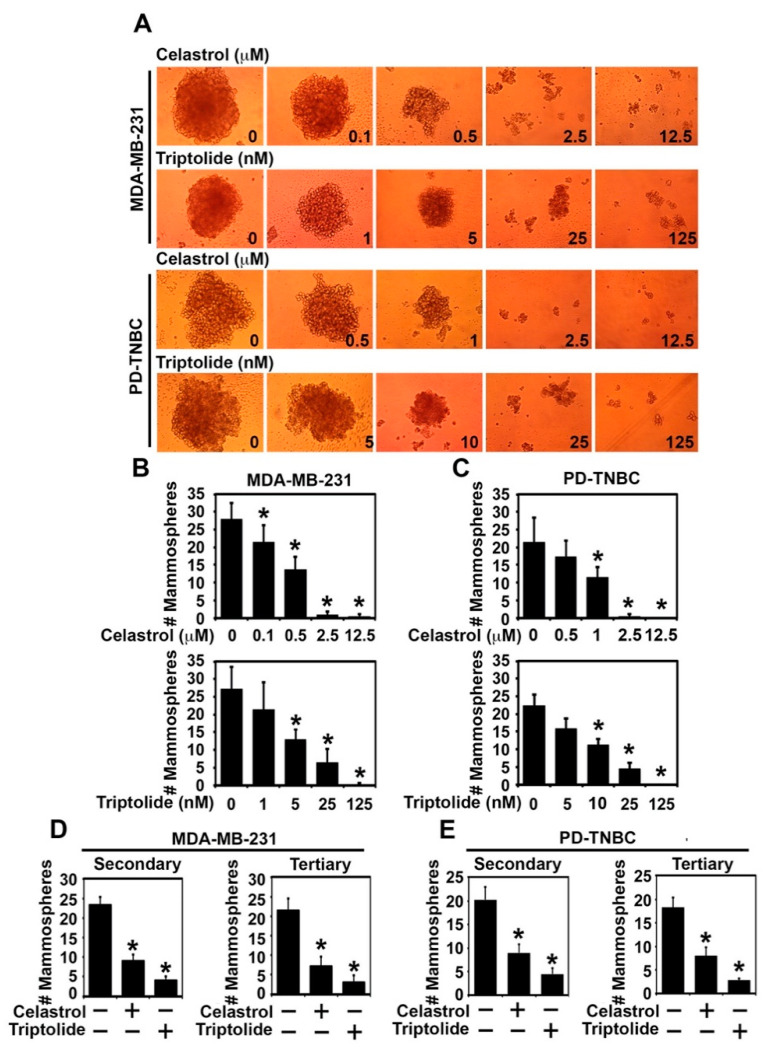
Celastrol and triptolide reduce mammosphere growth and number in TNBC cells. A and B) MDA-MB-231 and PD-TNBC cells were treated with celastrol (0–12.5 µM) and triptolide (0–125 nM). (**A**) Representative images show reduction in mammosphere size. (**B**,**C**) Total number (#) of primary mammospheres was reduced in a dose-dependent manner following celastrol and triptolide treatment when compare to control. * *p* < 0.05 when compared with control. (**D**,**E**) Primary mammospheres of MDA-MB-231, PD-TNBC cells treated with celastrol (0.5 µM) and triptolide (5 nM) were first treated with celastrol (1µm) and triptolide (10 nm), and subsequently used to grow secondary and tertiary mammospheres without drug treatment. * *p* < 0.05 when compared with control.

Standard 2D monolayer cultures have been the mainstay technique in traditional cultures for studying the biology of cancer cells. However, this does not represent the natural physiological environment that is observed in the tumors in vivo. Conversely, three-dimensional (3D) spheroid cultures gown in ultra-low conditions have a more physiological similarity to an in vivo tumor environment [30]. Hence, spheroid cultures have been used to demonstrate their functional role in tissue development and regeneration [31,32]. Hence, we plated two TNBC cell lines, MDA-MB-231 and BT20, in ultra-low culture plates in mammosphere medium and then treated the cultures with increasing doses of celastrol (0–12.5 µM) and triptolide (0–125 nM) for 72 h. There was a dose-dependent reduction in mammosphere formation with both celastrol and triptolide (Figure 1A, Supplementary Figure S1A). More importantly, there was a near 50% reduction in mammosphere when cells were treated with 500 nM celastrol or 5 nM triptolide. This was observed for both MDA-MB-231 and BT20 cells (Figure 1B, Supplementary Figure S1B). To confirm that the effect seen in the two cell lines is not a result of having adapted to the 2D culture conditions, we also developed a primary cell line from a patient tissue (PD-TNBC). Similar results were obtained when the cells were treated with celastrol and triptolide. Significant inhibition of mammosphere formation was observed with 1 µm celastrol and 10 nM triptolide when compared to control (Figure 1A,C). To check the long-term drug effect of the compounds, we also performed secondary and tertiary mammosphere cultures. Here we took the isolated mammosphere cells treated with 0.5 µM celastrol and 5 nM triptolide for MDA-MB-231, BT20, and 1 µM and 10 nM for celastrol and triptolide for PD-TNBC cells. Compounds were not added in the secondary and tertiary spheroid cultures. Again, all three cells showed reduced mammosphere growth in secondary and tertiary cultures (Figure 1D,E and Supplementary Figure S1C). To determine the effect of celastrol and triptolide on non-transformed cells, we incubated the compounds in normal non-tumorigenic breast epithelial cells, MCF10A. Neither celastrol nor triptolide demonstrated suppressed spheroid formation even at doses up to 5 mM celastrol and 50 nM triptolide (Supplementary Figure S1A,B). Moreover, triptolide demonstrated higher efficiency in inhibiting mammosphere growth when compared to celastrol in all three cell lines. Nevertheless, since no compound was added in the secondary and tertiary cultures and yet there was reduced mammosphere formation, this suggests that celastrol and triptolide are potent inhibitors of breast cancer stem cells.

## 4. Celastrol and Triptolide Affect the Stem Cell-Like Properties

**Figure 2:**
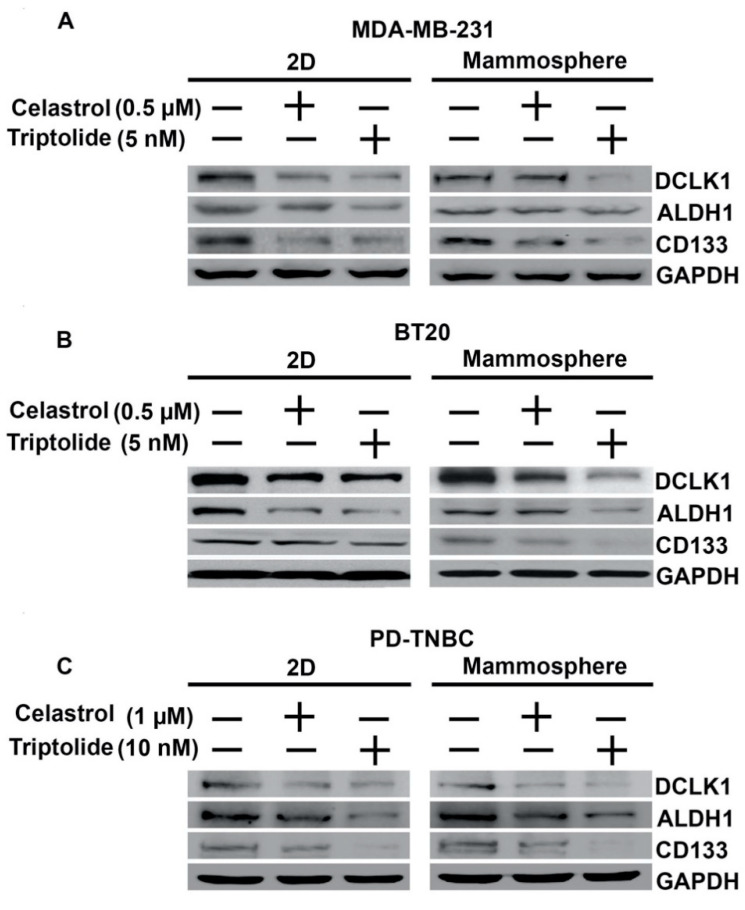
Celastrol and Triptolide attenuate expression of stem cell markers in TNBC cells. The two compounds inhibit cancer stem cells marker, DCLK1, ALDH1, and CD133 protein expression as compared to control, untreated cells in both 2D culture (left panel) and mammosphere cultures (right panel). (**A**) MDA-MB-231, (**B**) BT20, and (**C**) PD-TNBC.

Within a tumor, there are a variety of cell types that include CSCs and differentiated cancer cells. This is believed to complicate cancer treatment. Markers used for the identification of breast cancer stem cells include CD44, CD24, CD133, ALDH1, Lgr5, and DCLK1 [6,7]. We first determined the effects of celastrol and triptolide on DCLK1, ALDH1, and CD133 expression using western blot in the three cell lines. To determine if expression was different in 2D and mammosphere cultures, we performed studies in both culture conditions, treating them with celastrol and triptolide for 48 h. Western blot analyses of lysates showed a reduction in protein expression of DCLK1, ALDH1, and CD133 in both 2D and mammosphere cultures after celastrol and triptolide treatment when compared to the control in MDA-MB-231, BT20, and PD-TNBC cells (Figure 2). To further confirm these results, we performed flow cytometry for DCLK1, ALDH1, and CD133. In the control, untreated cells, all three cell lines showed significantly higher numbers of DCLK1+, ALDH+, and CD133+ cells in the mammospheres when compared to 2D cultures (Table 2). There was at least a 2–2.5-fold increase in DCLK1+ and ALDH1+ cells in the mammospheres, but a slightly smaller increase in CD133 + cells. However, following treatment with celastrol or triptolide, there was a reduction in the number of cells expressing these proteins in both 2D and mammosphere cultures. These data suggest that the two compounds suppress CSCs as assessed by the three stem cell protein markers.

## 5. Celastrol and Triptolide Inhibit Notch Signaling Pathway

**Figure 3:**
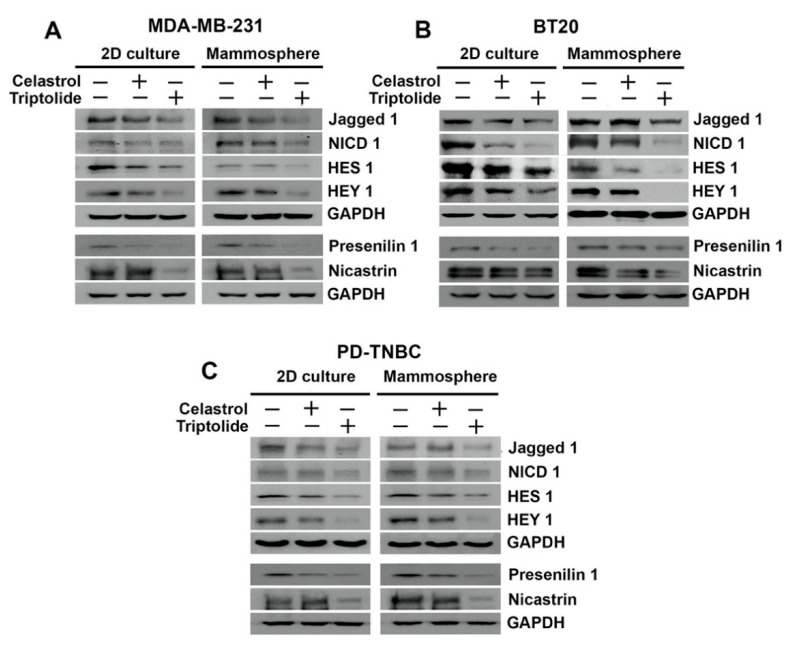
HSP90 inhibitors affect notch signaling in TNBC cells. Celastrol and triptolide inhibited notch signaling by decreasing the expression level of Notch ligand, Jagged-1, NICD 1, and Notch downstream target proteins HES1 and HEY1 in TNBC cells. Left panel shows blots from cells grown in 2D cell and right panel shows those from mammospheres. (**A**) MDA-MB-231 cells, (**B**) BT20 cells, and (**C**) PD-TNBC cells.

The Notch signaling pathway is a fundamental, conserved program that is important for cell–cell communication and controls various functions including timely cell lineage specification and differentiation [33]. Moreover, Notch signaling has been shown to promote resistance to targeted or cytotoxic therapies by enriching a small population of cancer stem cells [34]. There are four cell surface Notch receptors, Notch 1–4, and five ligands, Jagged 1, Jagged 2, Delta-like-1 (Dll 1), Dll 3, and Dll 4 [35]. When a ligand such as Jagged1 from the one cell binds to one of the Notch receptors in the accepting cell, it results in the triggering of a series of cleavages, ultimately releasing the intracellular domain (NICD). Subsequently, NICD translocates to the nucleus where it binds to its partner proteins to activate a stemness-related gene transcription program [34,35]. In breast cancers, Jagged 1 plays a significant role in promoting stem cell renewal and mammosphere formation [36]. Two downstream targets for Notch-driven transcriptional activation are Hairy and Enhancer of Split 1 (HES1) and Hairy/Enhancer-of-Split Related with YRPW Motif Protein 1 (HEY1). We first determined the effect of celastrol and triptolide on levels of four NICD protein in 2D and mammosphere culture in BT20 cells. We observed an isolated reduction of NICD1 after celastrol and triptolide treatment, while NICD2, NICD3, and NICD4 were not affected (Supplementary Figure S2). In addition to NICD1, there was also a reduction in the expression of Jagged 1 in both 2D and mammosphere cultures treated with celastrol and triptolide in the three cell lines (Figure 3). Moreover, there was a reduction in the expression of both HES1 and HEY1 following treatment with celastrol and triptolide. Together, these data suggest that both the compounds inhibit the Notch 1-signaling pathway.

**Figure 4:**
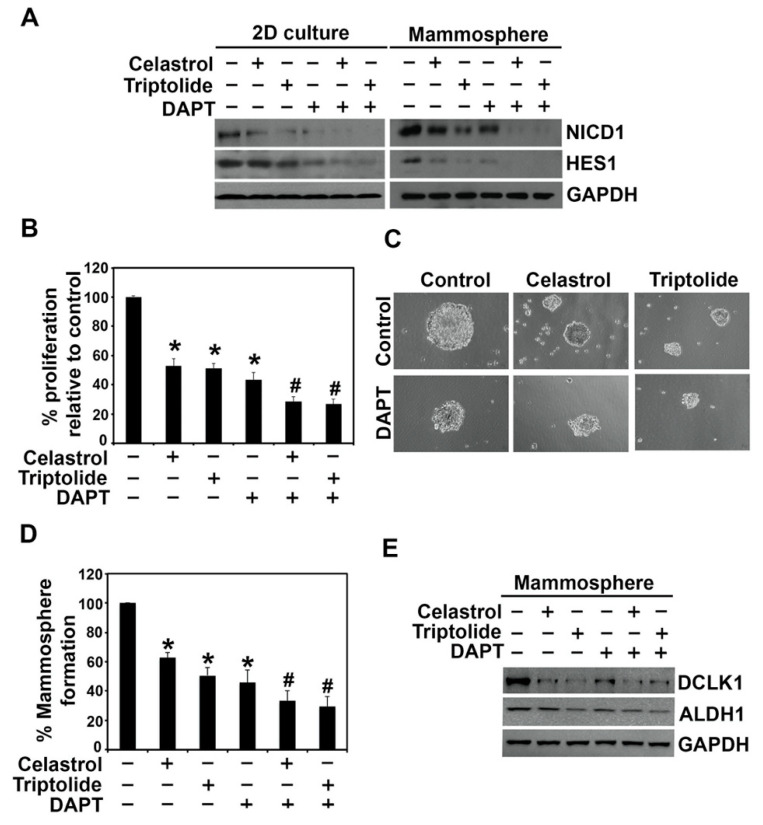
γ-secretase inhibitor, DAPT, altered the celastrol and triptolide-induced suppression of notch signaling in TNBC cells. (**A**) Western blot analysis of BT20 cells treated with DAPT, celastrol, and triptolide for 48 h. NICD1 and HES1 protein expression were suppressed in celastrol and triptolide treatment and further decreased in combination with DAPT. (**B**) Cell proliferation was significantly inhibited following treatment with the compounds and further reduced in combination of DAPT. Each value represents mean ± SD of four separate estimations, *n* = 3. * *p* < 0.05 when compared with control; # *p* < 0.05 when compared with DAPT control. (**C**,**D**) DAPT treatment alone reduces the mammosphere formation when compared to the control, and a combination with celastrol and triptolide shows further reduction in mammosphere growth. Each value represents mean ± SD of four separate estimations, *n* = 3. * *p* < 0.05 when compared with control; # *p* < 0.05 when compared with DAPT control. (**E**) Expression of stem cell marker proteins DCLK1 and ALDH1 is reduced following DAPT treatment. Combination treatment with celastrol or triptolide further decreases the expression of these proteins in mammospheres.

A pair of proteolytic cleavage events are essential for the release of NICD, starting with the first event at the extracellular domain followed by a second event that occurs inside the plasma membrane. The second cleavage event is catalyzed by the γ-secretase complex [37,38]. To address whether celastrol and triptolide affected the γ-secretase complex proteins, presenilin 1 and nicastrin, we performed western blot analyses. In all three cell lines, we observed that expression of both presenilin 1 and nicastrin were reduced in cells treated with the compounds (Figure 3). To further confirm the role of the γ-secretase complex in regulating stemness and Notch 1 activation, we treated BT20 cells with DAPT, a γ-secretase inhibitor. We chose BT20 cells for the Notch 1-related studies because of its high transfection efficiency. While DAPT alone decreased the levels of cleaved Notch1 receptor in BT20 cells, the combination of DAPT with celastrol or triptolide resulted in complete suppression of NICD1 and its target protein HES1 (Figure 4A). Moreover, while cells treated with DAPT alone showed reduced cell proliferation (Figure 4B) and mammosphere growth (Figure 4C,D), there was an additive effect when DAPT treatment was combined with celastrol and triptolide. Given the effects on spheroid formation, we performed western blot analyses to detect stem cell marker expression. Following treatment with DAPT, celastrol, and triptolide, both DCLK1 and ALDH1 expression was reduced in BT20 cells when compared to control (Figure 4E).

**Figure 5:**
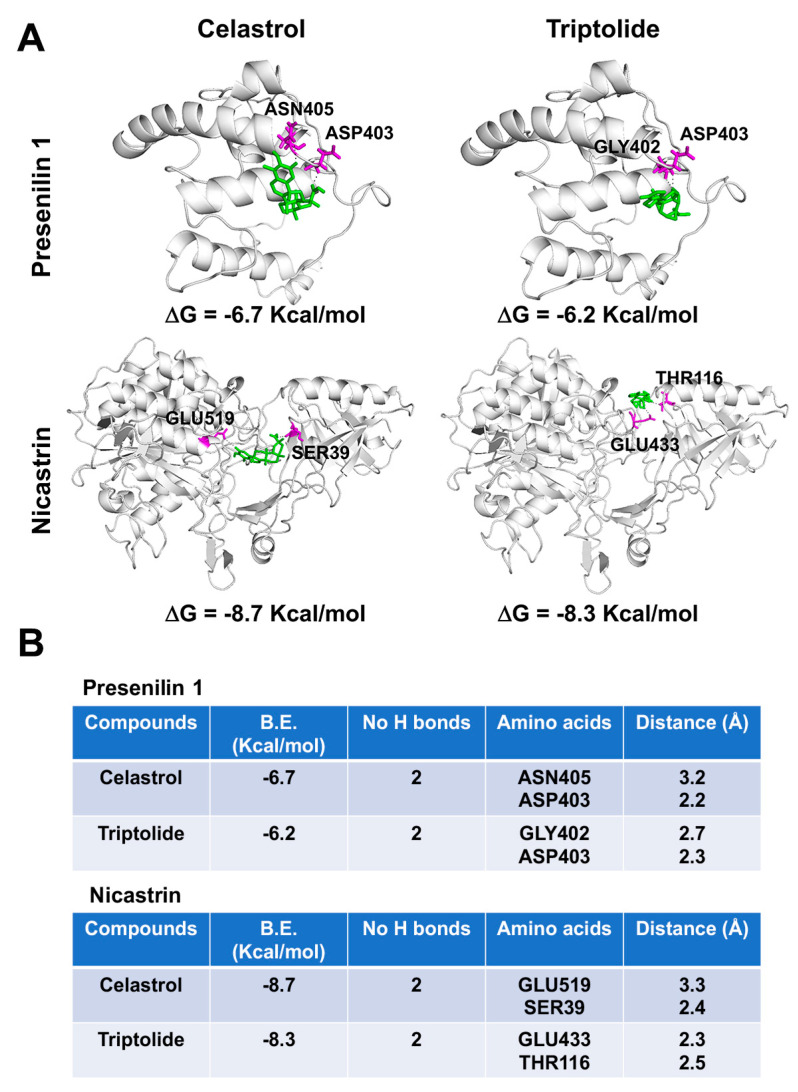
Celastrol and Triptolide bind to γ-secretase proteins presenilin 1 and nicastrin. (**A**) Binding of Celastrol and Triptolide in the protein cavity of presenilin 1 and nicastrin were assessed by molecular docking technique using Autodock vina software program. Celastrol and Triptolide bind to presenilin 1 and nicastrin with the binding energy (B.E.) of −6.7 and −7.2 Kcal/mol respectively, as well as bind to nicastrin with B.E. of −8.7 and −8.3 Kcal/mol respectively. Cartoon models are represented in the figure. (**B**) The docking results and consensus scores of Celastrol and Triptolide binding to presenilin 1 and nicastrin are summarized.

Since both compounds, celastrol and triptolide, affected intracellular Notch 1 levels, similar to that seen with DAPT, we performed molecular modeling of the compounds with the two proteins using the Autodock Vina software program. The docking data predicted that celastrol and triptolide bind to proteins with the binding energy of -6.7 and −6.2 Kcal/mol respectively in the case of presenilin 1 and −8.7 and −8.3 Kcal/mol in the case of nicastrin (Figure 5). Both compounds stabilized themselves by forming hydrogen bonds in the protein cavity. Celastrol forms hydrogen bonds with ASN405 (3.2 Å) and ASP403 (2.2 Å) within presenilin 1 protein cavity and with GLU519 (3.3 Å) and SER39 (2.4 Å) within nicastrin protein cavity. Similarly, triptolide forms hydrogen bonds with GLY402 (3.3 Å) and ASP403 (2.3 Å) within presenilin 1 protein cavity and with GLU433 (2.3 Å) and THR116 (2.5 Å) within nicastrin protein cavity (Figure 5B). The lower binding energy and stabilization through hydrogen bonding of celastrol and triptolide to both proteins suggested the tight binding and a probable mechanism of inhibition of the γ-secretase complex. In addition, presenilin 1 is reported to contain the two typical aspartyl residues (ASP275 and ASP385) that are critical for the active site on γ-secretase complex [32,33]. This region has been one of interest for designing g-secretase complex inhibitors for the treatment of Alzheimer’s Disease and cancers [32]. In our studies, we found that both compounds bind to Presenilin 1 in the proximity of ASP385, suggesting their inhibitory activity on γ-secretase complex.

**Figure 6:**
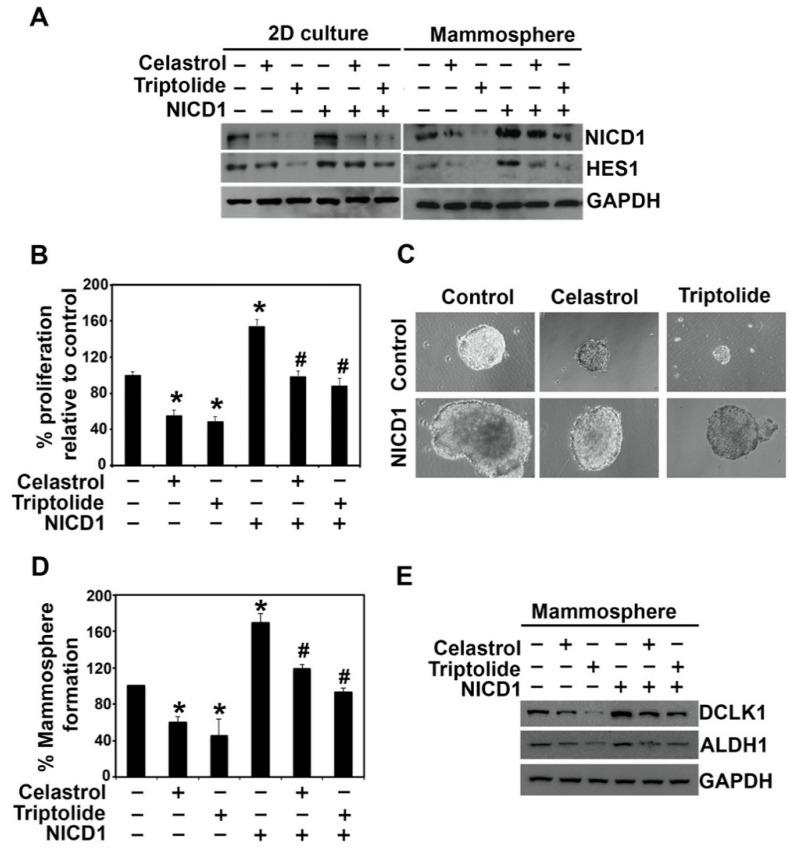
Overexpression of NICD1 partially protects from celastrol and triptolide treatment in BT20 cells. (**A**) NICD1 overexpression rescues cells from celastrol and triptolide-mediated suppression of NICD1 and HES1 expression. The cells were treated with the compounds for 48 h in cells overexpressing NICD1, and cell lysates were analyzed by western blotting. Increased protein expression of NICD1 and HES-1 was observed in the NICD-expressing cells when compared with vector controls. Celastrol and triptolide reduced the expression of NICD1 and HESI., but this was partially restored in NICD1 overexpressing cells. (**B**) NICD1-overexpressing BT20 cells overcome the suppression of cell growth in celastrol and triptolide. Each value represents mean ± SD of four separate estimations, *n* = 3. * *p* < 0.05 when compared with vector control; # *p* < 0.05 when compared with NICD1 overexpression control. (**C**,**D**) Mammosphere cultures show partial protection from celastrol and triptolide-induced spheroid growth in NICD1 overexpressing cells. Each value represents mean ± SD of four separate estimations, *n* = 3. * *p* < 0.05 when compared with vector control; # *p* < 0.05 when compared with NICD1 overexpression control. (**E**) DCLK1 and ALDH1 protein expression were decreased in celastrol and triptolide treatment but restored in NICD1 overexpressing cells after treatment.

To confirm that the effect of celastrol and triptolide is in part due to suppression of Notch 1 activation, we ectopically overexpressed NICD1 in BT20 cells, and subsequently treated it with celastrol and triptolide. As mentioned above, we chose BT20 cells because of their high transfection efficiency. While NICD1 overexpression increased the expression of HES1, it also protected the cells from celastrol and triptolide (Figure 6A). This was coupled with a significant increase in cell proliferation (Figure 6B) and mammosphere formation (Figure 6C,D). In addition, western blot analyses showed that DCLK1 and ALDH1 protein expression significantly increased in NICD-overexpressing BT20 mammospheres when compared to controls (Figure 6E). These results suggest that celastrol and triptolide inhibit stemness through the notch signaling pathway in TNBC cells. Together, these data suggest that both celastrol and triptolide suppress Notch signaling by inhibiting the expression of γ-secretase complex proteins, which reduces NICD1 activation, and ultimately decreases stem cell viability.

## 6. Discussion

TNBC is an aggressive subtype of breast cancer with no highly effective therapeutic options. Moreover, relapsing TNBC disease is a major problem where effective therapeutic interventions are even further limited. This study suggests HSP90 inhibitors may be a promising approach for TNBC, a subtype of breast cancer with poor prognosis that lacks approved targeted therapies [39,40,41]. Multiple HSP90 inhibitors have been developed such as geldanamycin derivatives and ganetespib, which have been shown to inhibit cancer cell growth in cell culture and in animal models. However, getting them to the clinic has been difficult in part due to significant toxicity. In this regard, we have demonstrated that celastrol and triptolide inhibit notch signaling to affect stemness in TNBC cells. Both compounds have been shown to exhibit similar pharmacological activities and have been defined as HSP90 inhibitors [14].

It is generally believed that CSCs promote tumor metastasis in addition to increasing recurrence risk and treatment resistance [42]. In our studies, we have observed that both celastrol and triptolide potently inhibit CSC viability based on the fact that the were able to suppress mammosphere formation. This is in agreement with recent studies suggesting that natural compounds such as geladanamycin, curcumin, and sulforaphane can target cancer stem cells [43,44,45]. The current studies are further strengthened by the observation that following treatment with these compounds, the CSC markers ALDH1 and CD133 showed decreased expression in cells grown in either 2D conditions or mammospheres. In addition, we have observed that DCLK1 expression is suppressed in these cells. This is an interesting observation because DCLK1 is increasingly being recognized as a stress-dependent reserve CSC marker in colon and other cancers [6,46]. In this regard, a recent study by Zhao et al. looked at developing an immunohistochemistry-based approach to classify TNBCs into molecular subtypes using androgen receptor (AR), CD8, FOXC1, and DCLK1 [47]. They observed that the mesenchymal subtype did not express AR, CD8 and FOXC1, but was DCLK1+. It would be interesting to determine whether expression of these markers can serve as a predictive marker of celastrol and triptolide effectiveness in specific TNBC subtypes.

Identifying cellular processes that activate CSC generation and maintenance is critical for developing novel modalities targeting these cells. Previous studies have shown various signaling pathways that play a role in stemness. Notch receptor activity is one such pathway that is upregulated in a variety of human cancer tissues [48,49,50]. In addition, activation of the pathway is important to preserve the mammary stem cells and may also play a role in breast cancer progression [35,36]. Furthermore, in mammosphere cultures, activation of the Notch pathway regulates self-renewal of the stem/progenitor cells [51]. Notch signaling can be affected at multiple points. Our studies with celastrol and triptolide point to inhibition of Notch1 receptor activation in part by targeting the γ-secretase complex proteins presenilin 1 and nicastrin. Loss of γ-secretase activity will suppress activation of Notch proteins, and this was confirmed by the DAPT studies. Molecular modeling studies also demonstrated potential high affinity interactions for the two compounds with presenilin 1 and nicastrin. Furthermore, ectopic expression of NICD1 alone was able to restore the stemness and expression of the CSC markers, further suggesting that a critical mechanism of action for celastrol and triptolide is inhibiting γ-secretase activity.

Another interesting outcome of these studies is that celastrol and triptolide inhibited expression of the ligand Jagged-1. BRCA1 has been shown to upregulate Jagged-1 and Notch 1 expression in breast cancers [52]. Moreover, inhibiting HSP90 using 17-AAG resulted in loss of BRCA1 by activating the ubiquitin-proteasome pathway [53]. Elevated expression of the Jagged-1 in patients with breast cancer correlated with poor overall survival [54,55,56]. Studies have demonstrated that Jagged-1 can also be cleaved at a site corresponding to its juxtamembrane region by ADAM17 [57]. Moreover, Jagged-1 can be cleaved within the intracellular region by γ-secretase, and the resultant intracellular domain can interact with Notch 1 intracellular domain to promoter its degradation by the ubiquitination-proteosome degradation pathway [58]. This suggests the complexity of this pathway and perturbation of its regulation can have profound effects on tumor progression and stemness. Given this fact, understanding the mechanism of reduction in Jagged-1 expression in response to celastrol and triptolide, and the implications for tumor growth needs to be further addressed. However, this does not discount the fact that the compounds can also affect the cleavage of the extracellular domain of Notch 1 protein.

In conclusion, the current studies demonstrate that HSP90 inhibitors celastrol and triptolide are potent suppressors of triple negative breast cancer cells, especially the stem cells. Additional studies are nevertheless required to further delineate how the compounds regulate Notch signaling and stemness in TNBC. In addition, in vivo studies are required in appropriate models to demonstrate the efficacy of the compounds in suppressing the relatively rare cancer stem cells in TNBC, while avoiding the toxicity seen in the clinical trials of HSP90 inhibitors tested thus far.

## Figures and Tables

**Table 1:** List of antibodies and dilution factor used for flow cytometry and western blot.

Antibody	Company	Catlog Number	Dilution (Flow Cytometry)	Dilution (Immunoblot)
Rabbit anti-DCLK1	Sigma Aldrich	SAB4200186	1-500	1-500
Mouse anti-CD133	MACS	130-092-395	1-50	1-50
Mouose anti-ALDH1	BD Biosciences	611194	1-200	1-500
Mouse anti-GAPDH	Santa Cruz	Sc51907	-	1-2000
Rabbit anti-Jagged1	Cell signalling	2155S	-	1-1000
Rabbit anti-Notch1	Cell signalling	4380S	-	1-1000
Rabbit anti-Notch2	Cell signalling	5732S	-	1-1000
Rabbit anti-Notch3	Cell signalling	5276S	-	1-1000
Rabbit anti-Notch4	Cell signalling	2423S	-	1-1000
Rabbit anti-Presenilin 1	Cell signalling	5643S	-	1-1000
Rabbit anti-Nicastrin	Cell signalling	5665S	-	1-1000
Rabbit anti-HES1	Abcam	Ab71559	-	1-1000
Rabbit anti-HEY1	Abcam	Ab22614	-	1-1000

**Table 2:** Celastrol and triptolide affect CSCs in TNBC cells compared with 2D vs. primary mammosphere. Mammosphere culture shows a greater number of DCLK1, ALDH1, and CD133 positive cells when compared to the control in MDA-MB-231, BT20, and PD-TNBC cells. (**A**,**B**) Triptolide-treated mammosphere shows greater reduction in DCLK1 and ALDH1 positive cells, but not in CD133 positive cells when compared to 2D culture. (**C**) Celastrol and triptolide show no difference in DCLK1 and CD133 positive cells between 2D and mammosphere culture. ALDH1 positive cells show a significant decrease in triptolide-treated mammosphere when compared to 2D culture.

A. MDA-MB-231			
**2D culture**	**DCLK1 (%)**	**ALDH1 (%)**	**CD133 (%)**
**Control**	7.3 ± 0.8	4.6 ± 1.3	11.6 ± 1.1
**Celastrol**	4.0 ± 0.5	3.2 ± 0.4	3.1 ± 0.7
**Triptolide**	3.3 ± 0.8	2.0 ± 0.7	2.8 ± 0.3
			
**Mammosphere**	**DCLK1 (%)**	**ALDH1 (%)**	**CD133 (%)**
**Control**	14.5 ± 2.4	12.1 ± 1.3	17.5 ± 1.9
**Celastrol**	7.0 ± 1.8	7.8 ± 0.9	9.4 ± 0.9
**Triptolide**	2.5 ± 0.7	2.4 ± 0.9	8.6 ± 2.4
			
**B. BT20**			
**2D culture**	**DCLK1 (%)**	**ALDH1 (%)**	**CD133 (%)**
**Control**	7.6 ± 0.8	7.4 ± 0.7	22.3 ± 2.9
**Celastrol**	5.4 ± 0.6	4.6 ± 0.6	11.4 ± 0.7
**Triptolide**	4.0 ± 0.8	3.9 ± 0.5	8.8 ± 0.9
			
**Mammosphere**	**DCLK1 (%)**	**ALDH1 (%)**	**CD133 (%)**
**Control**	19.1 ± 1.6	18.6 ± 1.1	29.3 ± 2.5
**Celastrol**	11.5 ± 0.6	14.5 ± 2.3	22.0 ± 6.4
**Triptolide**	6.0 ± 1.3	7.2 ± 0.5	14.8 ± 2.2
			
**C. PD-TNBC**			
**2D culture**	**DCLK1 (%)**	**ALDH1 (%)**	**CD133 (%)**
**Control**	5.4 ± 0.9	5.3 ± 0.7	14.5 ± 3.1
**Celastrol**	3.3 ± 0.5	3.6 ± 0.8	9.0 ± 1.5
**Triptolide**	1.5 ± 0.4	2.7 ± 0.3	4.7 ± 0.9
			
**Mammosphere**	**DCLK1 (%)**	**ALDH1 (%)**	**CD133 (%)**
**Control**	12.4 ± 1.8	12.1 ± 1.0	20.6 ± 3.1
**Celastrol**	7.6 ± 1.1	7.4 ± 0.7	10.5 ± 1.1
**Triptolide**	2.9 ± 1.1	2.6 ± 1.3	7.5 ± 0.8

## Data Availability

All raw data supporting the results of the present study can be obtained from the corresponding author upon reasonable request.

## Supplementary Materials

The following are available online at https://www.mdpi.com/article/10.3390/biomedicines9050482/s1, Figure S1: HSP90 inhibitors inhibit BT20 mammosphere growth. (**A**) BT20 cells were treated with celastrol (0–12.5 µM) and triptolide (0–125 nM) for 72 h. MCF10 A cells were incubated with increasing concentrations of celastrol (0–5 mM) and triptolide (0–50 nM) for 72 hrs. Representative images show reduction in the size and number of primary mammospheres in a dose-dependent manner following treatment when compared to the control. (**B**) Data from three independent primary mammosphere experiments show a dose-dependent decrease in the number of mammospheres following treatment with compounds. * *p* < 0.05 when compared with the control. (**C**) Primary mammospheres from BT20 cells treated with celastrol (0.5 µM) and triptolide (5 nM) were used for secondary and tertiary mammospheres in the absence of a drug. * *p* < 0.05 when compared with the control. Overall, decreases in percentages of secondary and tertiary mammosphere were compared with primary culture. Figure S2: Western blot showing differential expression of NICD1-4 isoforms in BT20 cells. Moreover, celastrol and triptolide inhibited NICD1 activation in both 2D and mammosphere cultures, but not that of NICD2, NICD3, or NICD4.

## Abbreviations

HSP90	Heat Shock Protein-90
TNBC	Triple Negative Breast Cancer
PD-TNBC	Patient derived-TNBC
DCLK1	Doublecortin Like Kinase-1
ALDH1	Aldehydedehydrogenase
NICD1	Notch Intracellular Domain-1
CSC	Cancer Stem Cell
