# Supplementary material for: Celastrol and Triptolide Suppress Stemness in Triple Negative Breast Cancer: Notch as a Therapeutic Target for Stem Cells
Source: Biomedicines. 2021 Apr 28;9(5):482. doi: 10.3390/biomedicines9050482 (PMC8146582; doi:10.3390/biomedicines9050482)
Supplement: Supplementary file 1 [file biomedicines-09-00482-s001.zip › biomedicines-1163651-supplementary.pdf]

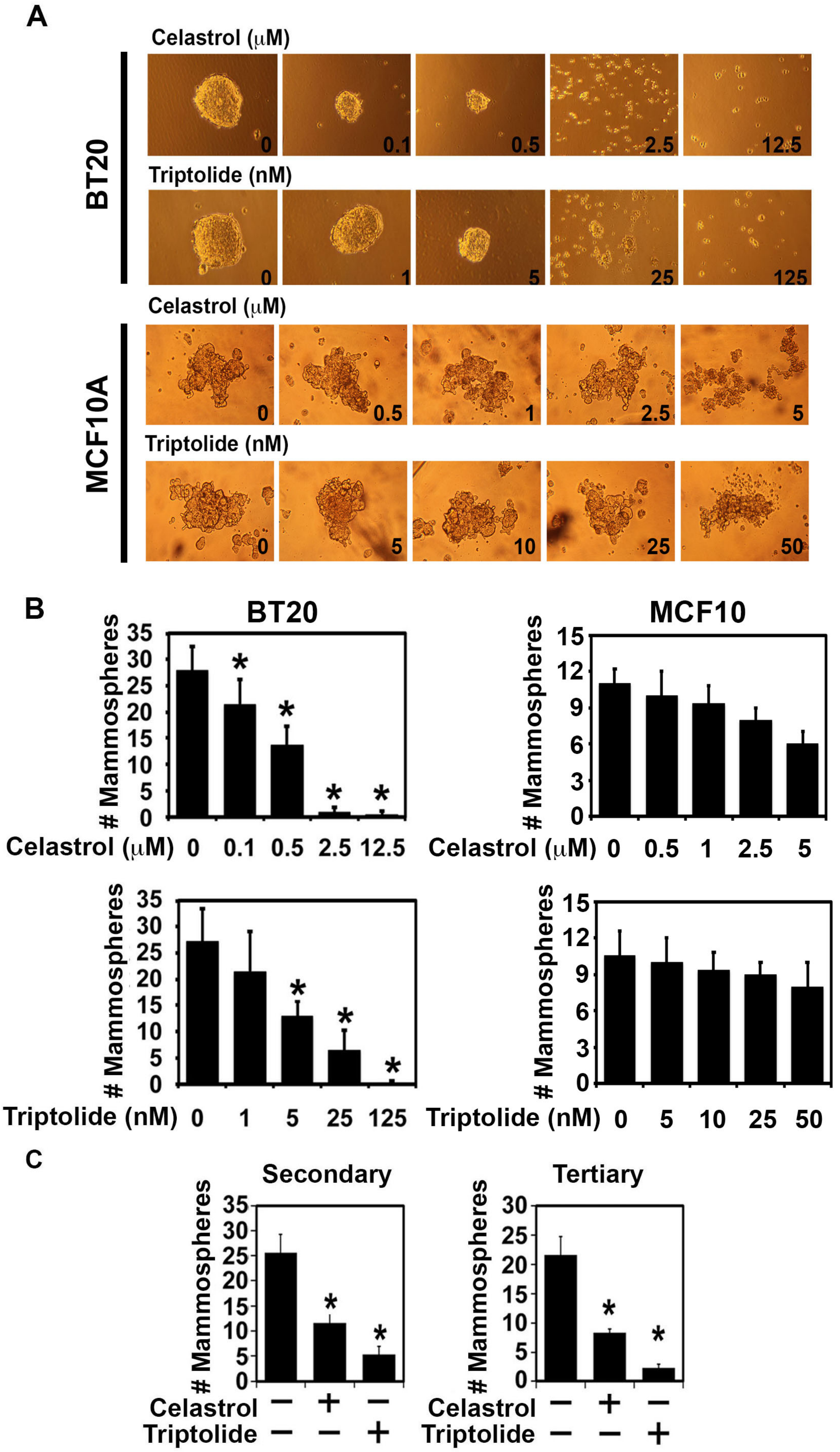
A
Celastrol (μM)
BT20
0
0.1
0.5
2.5
12.5
Triptolide (nM)
0
1
5
25
125
Celastrol (μM)
MCF10A
0
0.5
1
2.5
5
Triptolide (nM)
0
5
10
25
50
B
BT20
Mammospheres
Celastrol (μM) 0 0.1 0.5 2.5 12.5
MCF10
Mammospheres
Celastrol (μM) 0 0.5 1 2.5 5
Mammospheres
Triptolide (nM) 0 1 5 25 125
Mammospheres
Triptolide (nM) 0 5 10 25 50
C
Secondary
Mammospheres
Celastrol − + −
Triptolide − − +
Tertiary
Mammospheres
Celastrol − + −
Triptolide − − +

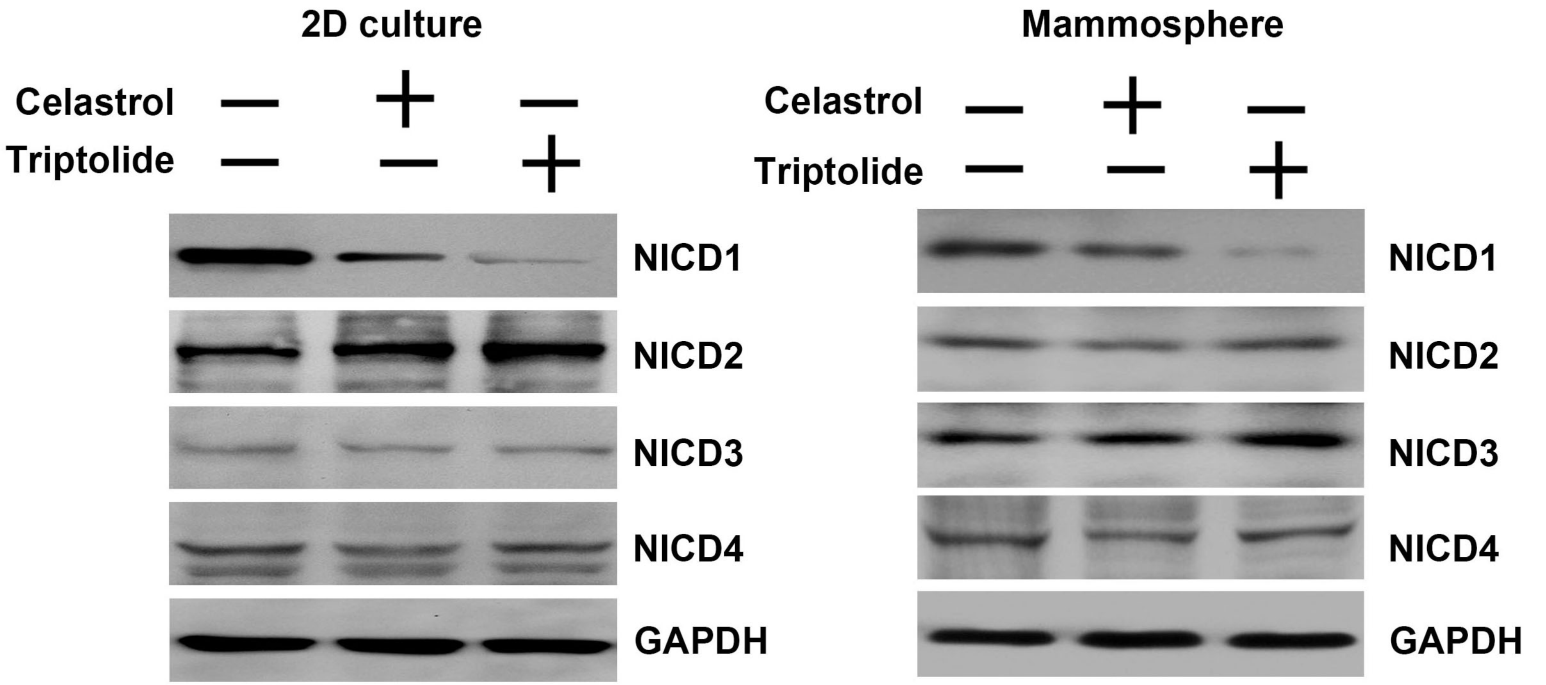

2D culture
Mammosphere
Celastrol
Triptolide
—
+
NICD1
NICD2
NICD3
NICD4
GAPDH
